# Tele-Drama Therapy for Older Adults With Constricted Life-Space Mobility: A Randomized Controlled Trial

**DOI:** 10.1093/geroni/igaf122.1808

**Published:** 2025-12-31

**Authors:** Talia Elkarif, Shoshi Keisari, Hod Orkibi

**Affiliations:** University of Haifa, Haifa, HaZafon, Israel; University of Haifa, Haifa, HaZafon, Israel; University of Haifa, Haifa, HaZafon, Israel

## Abstract

Drama therapy is a mental health profession that aims to promote well-being by systematically using dramatic tools and techniques, such as storytelling, dramatic improvisation, and role-play. Research on drama therapy demonstrates its effectiveness in enhancing the mental health and well-being of older adults. Ensuring access to such interventions for older adults with constricted life-space mobility is particularly important, as they are at greater risk for social isolation and poor psychological well-being. The current mixed-methods, randomized controlled trial (RCT), aimed to explore the transition to group tele-drama therapy for older adults with constricted life-space mobility. The quantitative arm of the study examined the impact of a 12-week tele-drama therapy intervention on mental health indices, and the relationship between in-session change factors and changes in outcome measures. The qualitative arm of the study explored the experiences of participants, drama therapists and community coordinators. A total of 111 older adults (aged 63-102) with constricted life-space mobility were randomly assigned to treatment or control groups. The intervention, integrating life review therapy and drama therapy techniques, was delivered via Uniper Care, a social platform with accessible technology designed for the aging population. Findings revealed a significant Time X Group interaction, demonstrating the intervention’s efficacy in reducing depressive symptoms and enhancing social connectedness, well-being, and personal growth. Thematic analysis of semi-structured interviews helped account for these quantitative findings, offering insights into treatment acceptability. These findings point to tele-drama therapy as an effective intervention for older adults with constricted life-space mobility, with implications for broader implementation.

